# Cardiovascular Pharmacology: Contemporary Physiologic and Scientific Approaches to Cardiovascular Disease Prevention and Treatment

**DOI:** 10.14797/mdcvj.1184

**Published:** 2022-12-06

**Authors:** James B. Young, Albert E. Raizner

**Affiliations:** 1 Cleveland Clinic Lerner College of Medicine of Case Western Reserve University, Cleveland, Ohio, US; 2 Houston Methodist DeBakey Heart & Vascular Center, Baylor College of Medicine, and Interventional Cardiology Associates, Houston, Texas, US

**Keywords:** cardiovascular pharmacology, therapeutics

Previous issues of the *Methodist DeBakey Cardiovascular Journal* have featured themes that include therapies for pulmonary hypertension, cardiovascular disease (CVD) prevention, cardiac arrhythmias, heart failure, and diabetes, among others. Robust clinical trial data on new pharmacologic therapies have driven professional societies to establish consensus guidelines for treating the spectrum of cardiovascular diseases.

In fact, medical care has undergone a dramatic paradigm shift due to therapeutic innovation and a better overall understanding of pharmacology. Cardiovascular pharmacology, in particular, is at the root of many if not most guideline recommendations regarding drug dispensation. In just 50 years, therapeutic approaches to heart failure, hypertension, pulmonary hypertension, coronary heart disease, atherosclerosis, infiltrative cardiomyopathies, and arrhythmias have challenged clinicians to pay close attention to the constant developments with the goal of decreasing morbidity and helping patients live a better quality of life.

With this in mind, we have developed this issue with a focus on cardiovascular pharmacology. As clinicians, we see how drug therapies can trigger events both beneficial and detrimental—producing negative reactions, ameliorating symptoms, and at times even curing disease. Today’s therapeutic arsenal encompasses hundreds of new drugs, and the pace with which new cardiovascular agents are given FDA approval can confuse even the most skilled physicians.

We aim to update our readers on several areas of progress in cardiovascular therapeutics, keeping in mind that the latest therapeutic guidelines for the most vexing conditions offer important opportunities to look to the future.

We kick off this issue with a condition encountered in all clinical practices: essential hypertension. Hypertension is one of the leading causes of mortality and disability worldwide and also one of the most treatable. In fact, just a 20-mm Hg decrease in systolic or 10-mm Hg decrease in diastolic blood pressure can dramatically decrease the risk of fatal cardiovascular events. Drs. Khurram Nasir, Behnam Heidari, and Eleonora Avenatti discuss candidates for hypertension treatment, therapies with the strongest supporting data, and studies showing how different antihypertensive medications have similar efficacy in treating hypertension and preventing its complications, with minimal differences between drug classes and between generic versus brand names.

Next we explore the various medical therapies for heart failure with preserved ejection fraction (HFpEF), an elusive disease that has long been challenged by the lack of both a unified definition and proven therapies. Drs. Barry Trachtenberg, Sara Varnado, and Hyeon-Ju Ryoo Ali discuss evidence-based options available to support the management of HFpEF and highlight the newest class of SGLT2 inhibitors, which are having dramatic effects across a wide array of cardiovascular patients, including those with HFpEF.

We then pivot to cardiac amyloidosis (ATTR), another elusive disease that can lead to heart failure, conduction disease, and arrhythmias. Despite improvements in noninvasive diagnostic tools, patients too often present with advanced disease, yet therapies for ATTR are most effective when administered before major symptoms appear. Drs. Jerry Estep, Trejeeve Martyn, Mazen Hanna, and Andres Carmona Rubio review the different forms of cardiac amyloidosis, the problem of missed or late diagnoses, how to develop clinical suspicion for ATTR, and opportunities for earlier identification and treatment.

From there, we delve into the evolution of glucocentric drugs in cardiovascular disease protection and heart failure. Even with optimal control, a diagnosis of type 2 diabetes is almost synonymous with an impending cardiovascular event, yet evidence for cardiovascular outcomes with older-generation antihyperglycemic drugs is based on data from older trials that did not include prespecified cardiovascular end points. In their review, Drs. Javed Butler, Khawaja Talha, Gregg Fonarow, and Salim Virani evaluate the cardiovascular risk profile of common older- and newer-generation drugs and propose a future course for evaluating glucocentric drugs.

We then take an interesting detour with a video Q & A featuring Dr. Steven Nissen, one of the first physicians to link Vioxx and other COX-2 inhibitors to an increased risk of heart attacks and strokes. Since then, his research into the scientific integrity behind the testing of certain drug classes forced a paradigm shift with the FDA that changed the rubric for clinical testing and reporting. Hear Dr. Nissen’s account of how vetting one of the world’s best-selling drugs ultimately resulted in safer therapeutic options for diabetic patients. This is one Q&A you won’t want to miss!

After this, Dr. Miguel Valderrábano examines the future of antiarrhythmic drug therapy and ponders if procedures will eventually supplant drug therapies. Although targeting the membrane potential at first seemed a logical direction for antiarrhythmic therapy, drug classification was confusing and inconsistent, grouping drugs by phenomena rather than molecular targets, making this neither clinically relevant nor mechanistically precise. Dr. Valderrábano discusses both the historic failures and uses of antiarrhythmic drugs, the rise of catheter ablation and device therapies, and the promising role of neuromodulatory and gene therapies.

Drs. John Cooke and Keith Youker close this issue by examining another paradigm shift, this one sparked by mRNA therapeutics. Despite the havoc created by the COVID-19 pandemic, one positive outcome was the accelerated use of mRNA in vaccine production. While most of the emerging mRNA therapies are still in preclinical development, their application to cardiovascular disease is virtually limitless, with the potential to treat myocardial ischemia, heart failure, arrhythmias, hypercholesterolemia, and arterial occlusive diseases. The authors discuss the future of mRNA therapeutics for cardiovascular diseases, their use to enhance cell therapies, their ability to be personalized for individual patients, and the role of academic hospitals in advancing their generation and application.

Monitoring and predicting the success of a medication may seem, at times, a Sisyphean task. But the persistent study and testing of new and emerging cardiovascular drugs has led to many positive developments in patient care, including safer and effective treatments. We are grateful to our experts who have provided up-to-date reviews on the constantly changing world of CV pharmaceuticals, and we hope our readers find these topics interesting, useful, and applicable to their clinical practice. We encourage you to explore other related reviews on the journal’s website.

## Editor Biographies

The editorial team of the *Methodist DeBakey Cardiovascular Journal* expresses our thanks to Dr. James Young and Dr. Albert Raizner for their creativity, insight, and dedication in curating this issue on cardiovascular pharmacology.

## Albert E. Raizner, MD

**Figure:**
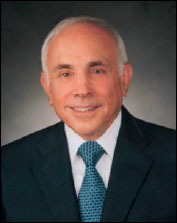


Dr. Raizner is vice-chairman of the Department of Cardiology at the Houston Methodist DeBakey Heart & Vascular Center, clinical professor at Baylor College of Medicine, and the senior partner of Interventional Cardiology Associates. He launched his cardiology career in Houston in 1972 when he was recruited to the faculty at Baylor College of Medicine. In 1979, he was appointed director of the Cardiac Catheterization Laboratory at Houston Methodist Hospital, a position he would hold for 25 years. In 2000, he helped found the Houston Methodist DeBakey Heart & Vascular Center and subsequently served as its first medical director.

Throughout his career, Dr. Raizner has been at the forefront of interventional cardiology, performing some of the first angioplasties and stent implantations in the country. His research is published in more than 200 scientific articles, 24 book chapters, and one book that contributed to the validation, approval, and use of stents in coronary arteries and led to methods to prevent restenosis. He has been instrumental in developing devices used in interventional cardiology and has been a principal investigator in dozens of clinical trials. He has shared his expertise serving on the Interventional Cardiology Board of the American Board of Internal Medicine, the Board of Trustees of the Society of Cardiac Angiography and Interventions, and as a fellow of numerous cardiology societies. He has been a consultant to NASA, the US Nuclear Regulatory Commission, and the US Department of Justice.

## James B. Young, MD

**Figure:**
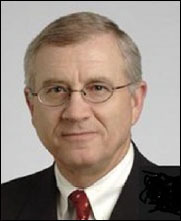


Dr. Young is an advanced heart failure and transplant cardiologist who did his undergraduate work at the University of Kansas and received his medical degree from Baylor College of Medicine in 1974. He completed his Internal Medicine residency in the Baylor Affiliated System and was Chief Medical Resident at Houston Methodist Hospital. His cardiology fellowship was also in the Baylor system. He joined the Baylor faculty in 1979 and was primarily stationed at Ben Taub General Hospital, where he became Chief of Cardiology. He subsequently became a tenured professor of medicine while moving to Houston Methodist Hospital as inaugural Clinical Coordinator and Scientific Director of Michael E. DeBakey’s Multiorgan Transplant Center. In 1995, he migrated to the Cleveland Clinic to create a section of Heart Failure and Cardiac Transplant Medicine. Roles he held in Cleveland included vice chair of the Department of Cardiovascular Medicine, chair of the Division of Medicine, chair of the Department of Endocrinology and Metabolism, dean of Cleveland Clinic Lerner College of Medicine of Case Western Reserve University, Chief Academic Officer, and director of Academic Affairs. He holds the George and Linda Kaufman Chair in the Heart, Vascular and Thoracic Surgery Institute.

Dr. Young’s academic interests include cardiac and multiorgan transplantation, mechanical circulatory assist devices, therapeutic clinical trials, and bridging basic science research to clinical applications. He has authored or coauthored more than 700 published manuscripts and several textbooks. He also has an interest and passion for medical humanities and is currently Section Editor of the *Methodist DeBakey Cardiovascular Journal* POET’S PEN feature.

